# Essential operating principles for tumor spheroid growth

**DOI:** 10.1186/1752-0509-2-110

**Published:** 2008-12-23

**Authors:** Jesse A Engelberg, Glen EP Ropella, C Anthony Hunt

**Affiliations:** 1 UCSF/UC Berkeley Joint Graduate Group in Bioengineering, University of California, San Francisco, CA, USA; 2 The Department of Bioengineering and Therapeutic Sciences, University of California, San Francisco, CA, USA

## Abstract

**Background:**

Our objective was to discover in silico axioms that are plausible representations of the operating principles realized during characteristic growth of EMT6/Ro mouse mammary tumor spheroids in culture. To reach that objective we engineered and iteratively falsified an agent-based analogue of EMT6 spheroid growth. EMT6 spheroids display consistent and predictable growth characteristics, implying that individual cell behaviors are tightly controlled and regulated. An approach to understanding how individual cell behaviors contribute to system behaviors is to discover a set of principles that enable abstract agents to exhibit closely analogous behaviors using only information available in an agent's immediate environment. We listed key attributes of EMT6 spheroid growth, which became our behavioral targets. Included were the development of a necrotic core surrounded by quiescent and proliferating cells, and growth data at two distinct levels of nutrient.

**Results:**

We then created an analogue made up of quasi-autonomous software agents and an abstract environment in which they could operate. The system was designed so that upon execution it could mimic EMT6 cells forming spheroids in culture. Each agent used an identical set of axiomatic operating principles. In sequence, we used the list of targeted attributes to falsify and revise these axioms, until the analogue exhibited behaviors and attributes that were within prespecified ranges of those targeted, thereby achieving a level of validation.

**Conclusion:**

The finalized analogue required nine axioms. We posit that the validated analogue's operating principles are reasonable representations of those utilized by EMT6/Ro cells during tumor spheroid development.

## Background

Extensive study of EMT6/Ro (hereafter EMT6) multicellular tumor spheroids grown in culture has provided useful insight into important aspects of tumor growth and tumor cell culture models. The behaviors of EMT6 cells in culture fall reliably within narrow ranges, as if cell behavior and thus the underlying mechanisms are tightly choreographed. Those actions can be thought of as being constrained and guided by a set of genetically specified biological operating principles. Can we discover and attribute a small, robust set of operating principles that combine to create the system level phenomena that characterize EMT6 growth *in vitro*? How can we represent and challenge those operating principles? What organization of the subcellular molecular biology enables the operating principles to emerge, and be sustained at the cellular level? Before addressing the last question, we need answers to the first two, which has been the objective of this project.

**Figure 1:**
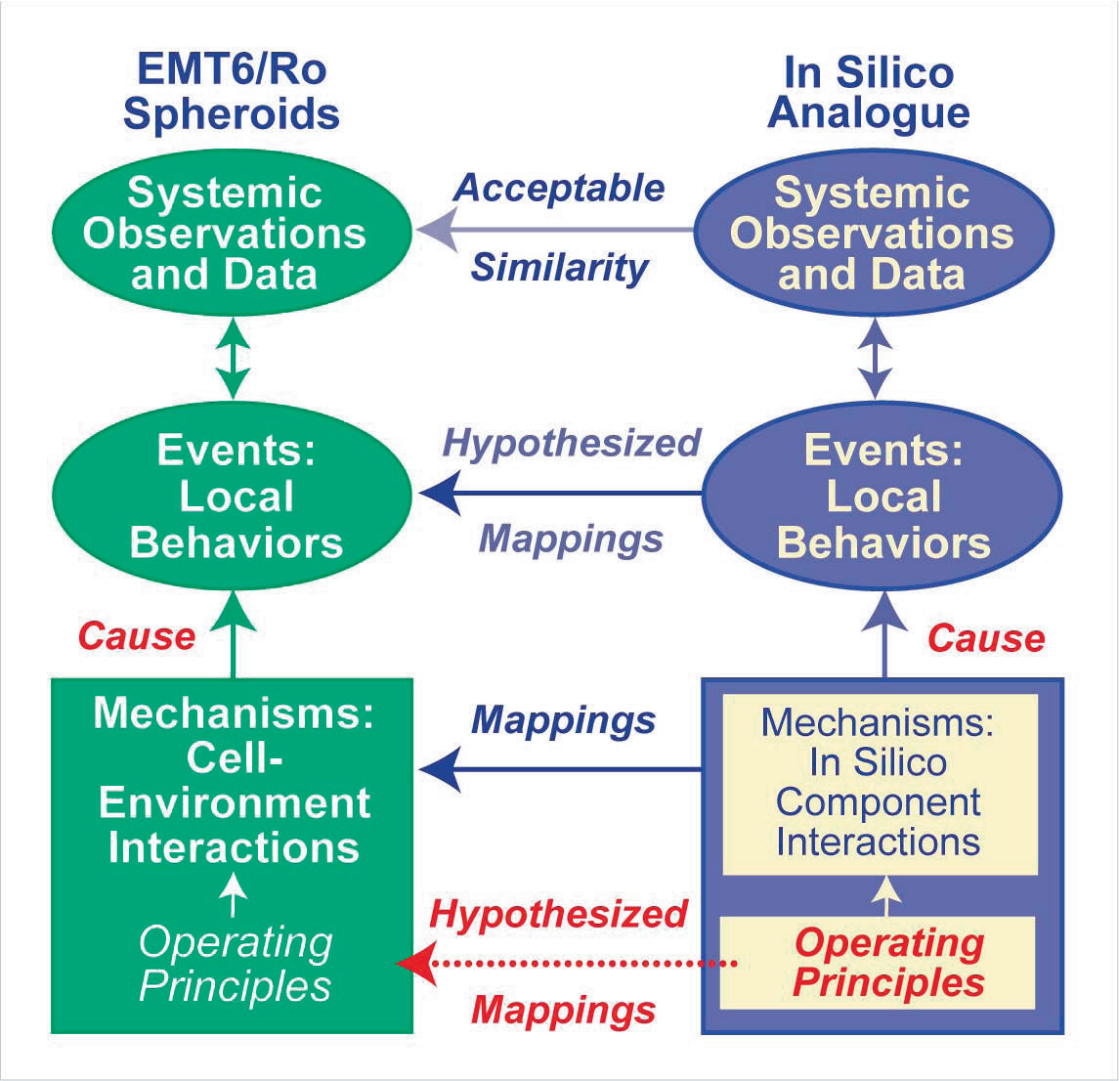
**Relationships between simulated multicellular tumor spheroids (SMS) and EMT6 spheroids**. An SMS is comprised of quasi-autonomous cell components interacting with adjacent cells and factors in their environment by adhering to a set of axiomatic operating principles. A clear mapping exists between SMS components and EMT6 counterparts. Following execution, the interacting components cause local and systemic behaviors. Measures of cell and system behaviors provide a set of attributes – the SMS phenotype. Validation was achieved when SMS attributes were measurably similar to a targeted set of EMT6 attributes. When that was accomplished, we could hypothesize that a semiquantitative mapping exists between in silico and *in vitro *events. We could also hypothesize that the set of axiomatic operating principles has a biological counterpart.

An approach to understanding how individual cell behaviors can contribute to a diverse set of system level attributes is to discover a set of simple yet sufficient principles that enable abstract, cell mimetic agents, using only locally available information, to exhibit behaviors closely analogous to cells in culture. For this context, we define a biological operating principle to be an abstract, inferential representation of an action within a reliably behaved cell system. To discover these principles, we created a quasi-autonomous computer analogue comprised of individual cell mimetic agents (CELLS) that adhered to a common, small set of axiomatic operating principles. We use axiom as commonly defined [1] and to emphasize that the analogue, unlike EMT6 cells in cultures, is a formal mathematical system and its execution is a form of deduction from the axioms within the analogue. Hereafter, we use AXIOM to emphasize that we refer only to the computational analogue. An AXIOM specified a behavior that depended on the local environment perceived by the CELL, given its internal state. Individual AXIOMS were implementations of in silico, axiomatic operating principles. Each axiomatic operating principle was derived from a postulated *in vitro *counterpart as described in Methods. The combined actions of an expanding population of CELLS, each adhering to the same set of operating principles, were sufficient to produce unique systemic behaviors. The system underwent several rounds of iterative refinement and parameter tuning. When measured, the resulting behaviors provided a set of systemic attributes that matched observed *in vitro *attributes closely for two different growth conditions. Once that was achieved, we could postulate that the axiomatic operating principles may have *in vitro *counterparts, as illustrated in Fig. 1. To date, such principles have been arrived at piecemeal by induction following experimentation. Experimental cell biology has been successful at discovering isolated cell level operating principles, but progress has been slow in providing a unified understanding of autonomous cellular behavior. We anticipated that iterative analogue refinement would lead to improved insight into cell level operating principles and plausible mappings to their biological counterparts. Even though the EMT6 cell line is tumor-derived, because it has proven stable and exhibits reliable behaviors, for the purposes of this research, we can treat EMT6 cells as being in a healthy, not diseased state.

Efforts to model tumor spheroid growth characteristics (see [2], two recent reviews [3,4], and references therein) have been extensive, informative, and successful. However, no one has proposed a cohesive set of cell level operating principles. Only recently has it become feasible to design and instantiate quasi-autonomous, cell mimetic analogues, [5-7] capable of exhibiting a rich phenotype of their own. The focus of most modeling and simulation efforts has not been in that direction. It has been primarily to provide precise, mostly mathematical descriptions of growth dynamics in terms of measured biochemical and physical factors combined with detailed descriptions of essential cell processes. The resulting models have been successful in explaining the time course and limits of spheroid growth in terms of nutrient depletion [8], increased acidity near the spheroid's center [9], and the dynamics of tumor spheroid metabolism [10]. Jiang et al. combined these features into a comprehensive model that separately considered each cell and spanned three levels of mechanistic resolution [2]. Other modeling efforts such as [11,12] have used hybrid mathematical and individual based approaches that have shown initially promising qualitative results.

The objective of this project has been different: we aimed to discover a somewhat minimalist set of essential axiomatic operating principles that would enable the system level growth characteristics produced by CELLS to match a targeted set of tumor spheroid growth attributes, based on measures of similarity. Given that constraint, we identified nine axiomatic operating principles. To narrow the scope and to achieve one of the key targeted attributes, we insisted that CELLS only acquire and use information available locally. We designed the system so that systemic properties were a consequence of only local CELL interactions. We made it easy to revise CELL AXIOMS so that we could sequentially expand the set of targeted attributes achieved. The targeted attributes achieved (Table 1) include those that are most characteristic of *in vitro *tumor spheroid growth: development of a stable size; a three-layered structure that maps to outer, proliferating cells and a core of inner, necrotic cells, with quiescent cells in between; the ability of cells to shed; and realistic growth curves under two different growth conditions. To achieve the targeted attributes, it was not necessary to simulate the release of growth inhibitory substances.

**Table 1:** Targeted attributes.

**Attribute**	**Description**
1	Cells consume resources, change state, proliferate, lose adhesion, die, shed, and move.
2	Cells proliferate throughout the duration of growth of the EMT6 spheroid.
3	Cells behave autonomously and locally.
4	The EMT6 spheroid develops an inner necrotic core, a middle quiescent layer, and an outer proliferating layer.
5	The EMT6 spheroid initially grows exponentially, then linearly, and then stabilizes.
6	The EMT6 spheroid has different growth characteristics at different levels of nutrient.
7	Necrosis onset occurs when the EMT6 spheroid has an area of roughly 0.2 mm^2 ^at high nutrient and 0.02 mm^2 ^at low nutrient.
8	The viable rim has a width of roughly 240 μm at high nutrient and 60 μm at low nutrient.
9	The measured initial doubling times are roughly 22 hours at high nutrient and 26 hours at low nutrient.
10	The mean error percentage between EMT6 spheroid and SMS growth is within 15% at high and low nutrient levels.

A cohesive set of operating principles (as distinct from isolated principles) can provide a framework into which more detailed, subcellular and molecular level information can be connected directly to system level phenotype. The plan was to work backward from a targeted set of *in vitro *observations of EMT6 cell and spheroid phenomena to a plausible set of analogue AXIOMS, which would be necessary and sufficient to generate in silico counterparts of the targeted phenomena. With that vision, this project has been motivated by three expectations: 1) Understanding hypothesized mechanisms *in vitro *would be facilitated by successfully building and studying analogous mechanisms in silico. 2) Achieving and refining validated analogues would offer a scientific, experimental approach to discovering and studying cohesive sets of operating principles. 3) Knowledge of axiomatic operating principles would facilitate exploration of their biological counterparts. This article reports on the design and implementation of the analogue, and the results of its execution.

## Results

**Figure 2:**
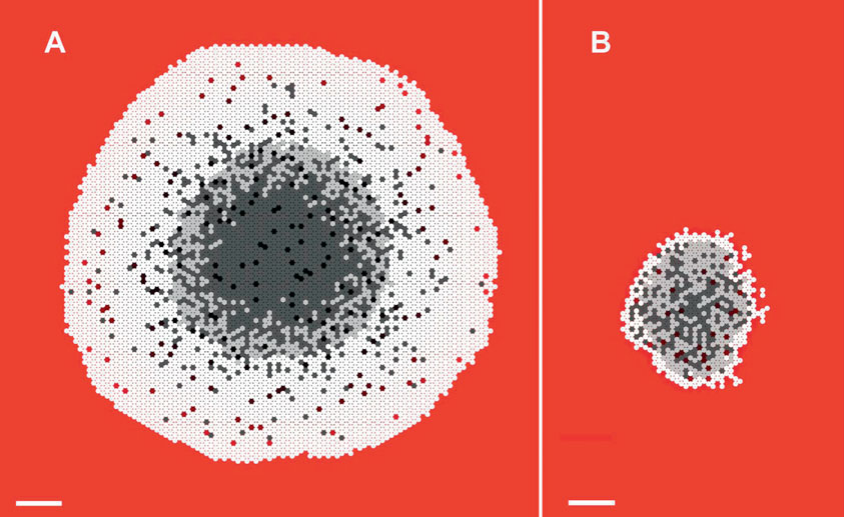
**SMS cross-sections at 17 DAYS**. Scale bar: 100 μm. Parameter values were those listed in Table 2. White circles: proliferating CELLS; light gray circles: quiescent CELLS; dark gray circles: NECROTIC CELLS. The background gradient (from red to black) represents NUTRIENT levels relative to the maximum value in red. (A) Growth occurred at high NUTRIENT, which maps to 0.28 mM oxygen and 16.5 mM glucose. (B) Growth occurred at low NUTRIENT, which maps to 0.08 mM oxygen and 0.8 mM glucose.

**Figure 3:**
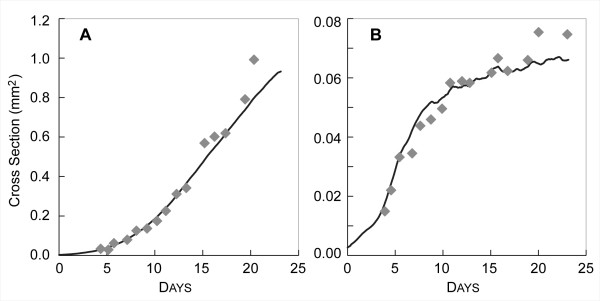
**EMT6 and SMS growth curves**. In vitro growth values (gray diamonds) were adapted from [13] by calculating spheroid diameters from measured volumes, assuming a circular cross-section. SMS values were obtained by specifying that CELL diameter maps to 10 μm, measuring the greatest X, Y extents, excluding isolated CELLS, and assuming a circular cross-section. Parameter values were those listed in Table 2. (A) SMS growth at high NUTRIENT. Values are means of ten runs. EMT6 spheroid values were from [13] at 0.28 mM oxygen and 16.5 mM glucose. (B) SMS growth was under low NUTRIENT. Values are means of ten runs. EMT6 spheroid values from [13] at 0.07 mM oxygen and 0.8 mM glucose.

To distinguish clearly in silico components and processes from corresponding components and processes within EMT6, we use SMALL CAPS when referring to the former. Variable names are in *italics*, and each is defined in the order it is introduced. CELL growth leads to formation of Simulated MULTICELLULAR Spheroids (SMS). Measurements of SMS attributes during execution mimic characteristics of EMT6 spheroid growth. Figure 2 shows an example qualitative measurement of the SMS as a two dimensional cross-section. It shows that SMS displayed the characteristic layered structure of EMT6 spheroids. The PROLIFERATING layer appears thicker than is often seen in EMT6 spheroids, but note that while the majority of CELLS in the VIABLE rim are in the PROLIFERATING state, only the CELLS on the outer layer of the SMS are actively creating new CELLS. Figure 3 shows that SMS growth curves were similar to reported EMT6 spheroid growth curves when CELLS used the parameters listed in Table 2 and the nine axiomatic operating principles listed in Table 3. AXIOM application was rigid in the sense that when a precondition was met, the appropriate AXIOM was always applied. AXIOMS 7 and 9 were stochastic. During a simulation cycle, a CELL could subsequently apply more than one AXIOM, such as 1, 4, 7, and 9 for a PROLIFERATING CELL or 3 and 5 for a NECROTIC CELL.

**Table 2:** Parameter names, values, units and sources.

**Parameter**	**In silico value**	***In vitro *value**	**Source**
Proliferating NUTRIENT critical level (*proNut*)	3.0 × 10^-3^	3.0 × 10^-19 ^mol/μm^3^	Tuned parameter
Quiescent NUTRIENT critical level (*quiNut*)	8.0 × 10^-4^	8.0 × 10^-20 ^mol/μm^3^	Tuned parameter
Proliferating CELL'S NUTRIENT uptake (*proConsumeRate*)	5.0 × 10^-4^/SEC	5.0 × 10^-17 ^mol/(cell s)	[17]
Quiescent CELL'S NUTRIENT uptake (*quiConsumeRate*)	1.0 × 10^-4^/SEC	1.0 × 10^-17 ^mol/(cell s)	Tuned parameter
Delay before dead CELL is removed (*removeDelay*)	3.6 × 10^4 ^SEC	1.8 × 10^4 ^s	[35]
Movement bias (*moveEmptyBias*)	1.0	--	Tuned parameter
Delay between CELL creation events (*prolifDelay*)	800 SEC	800 s	Tuned parameter
Proliferation bias (*proBias*)	2.25	--	Tuned parameter
NUTRIENT diffusivity (*diffusionRate*)	0.28*	105 μm^2^/s	[17]
Initial NUTRIENT concentration (*initialVal*)	0.165 or 0.008	16.5 mM or 0.8 mM	[27]
			
Time step	1.0 SEC	1.0 s	Calculated
CELL diameter	1 grid space	10 μm	[26]
Average cell cycle**	~4.24 × 10^4 ^SEC	~4.24 × 10^4 ^s	Calculated
Average time of removal after cell death**	1.8 × 10^4 ^SEC	1.8 × 10^4 ^s	Calculated

Sourced parameters were obtained from literature. Tuned parameters were adjusted to allow the analogue to reproduce *in vitro *data. Calculated parameters depended on sourced or tuned parameters. All parameters were fixed, except *initialVal*, which was set to high (equivalent to 0.28 mM oxygen and 16.5 mM glucose) or low (equivalent to 0.07 mM oxygen and 0.8 mM glucose) in different simulations.

* The value of *diffusionRate *is related to the simulation cycle and CELL diameter, as described within the text.

** The mean value observed during simulation.

**Table 3:** SMS AXIOMS.

**AXIOM**	**Environment**	**Action**	**Parameters used**	***In vitro *source**
1	NUTRIENT* > proNut*	Switch to PROLIFERATING state	*proNut*	Cell quiescence is regulated by the glucose and oxygen supply [19].
2	NUTRIENT <*quiNut*	Switch to NECROTIC state	*quiNut*	Cell death is regulated by the glucose and oxygen supply [19].
3	*quiNut *<NUTRIENT <*proNut*	Switch to QUIESCENT state	*proNut, quiNut*	Cell quiescence is regulated by the glucose and oxygen supply [19].
4	State = PROLIFERATING or QUIESCENT	Consume NUTRIENT equal to *proConsumeRate *or *quiConsumeRate*	*proConsumeRate, quiConsumeRate*	Cells consume oxygen and glucose at varied levels [17].
5	State = NECROTIC; *removeCounter *< 0	Remove CELL	*removeDelay*	Necrotic cells eventually break up and are consumed [35].
6	Inside CELL adjacent to empty space	Move into empty space		Cells move and mix with other cells within the spheroid [18].
7	Outside CELL adjacent to empty space	Move into empty space with prob. p_m_	*moveEmptyBias*	Cells move and mix with other cells within the spheroid [18].
8	Outside CELL with 0 neighbors	Randomly move in space		Cells can be shed from the exterior of the spheroid [21].
9	State = PROLIFERATING; *prolifCounter *< 0; CELL has empty neighbors	Create new CELL with prob. p_b_	*prolifDelay, proBias*	Cells create new cells within the SMS, causing it to increase in size [19].

In each time step a CELL will execute one or more of these AXIOMS based on the environment the CELL is exposed to and its internal variables. The AXIOMS are executed in the order specified within Additional file 1, Fig. S4. Random numbers for probability functions are generated from a uniform distribution in [0,1), except as noted in the text.

### In silico growth curves matched *in vitro *growth curves

For the parameter values listed in Table 2, SMS growth curves were quantitatively similar to those of EMT6/Ro spheroids for both high and low nutrient conditions. CELLS within an SMS proliferated initially at an exponential pace. Growth then slowed and became linear because only CELLS near the outer SMS rim could reproduce. The increase in cross-sectional area was linear until CELLULAR NECROSIS began. Thereafter, SMS growth rate began decreasing toward zero. A stable size was reached when CELL creation was balanced by CELL removal. Plots of SMS cross-sectional area over time (Fig. 3) closely mirrored EMT6 spheroid growth [13] for both high and low levels of NUTRIENT. For simplicity, as discussed under Methods, we conflated measured concentrations of glucose and oxygen, along with the other *in vitro *nutrients, and represented the entire collection using the factor NUTRIENT. High NUTRIENT level mapped to 16.5 mM glucose and 0.28 mM oxygen. Low NUTRIENT level mapped to 0.8 mM glucose and 0.07 mM oxygen. The reported coefficient of variation of mean cross-sectional EMT6 spheroid area between multiple *in vitro *experiments was roughly 29% at 7 days, increasing over time [14]. Given that, and the fact that EMT6 spheroids increase their size by many orders of magnitude during growth, we judged that having simulated values within 15% of referent values would be reasonable, and made that a targeted attribute (Table 1). The mean percent error between in silico and *in vitro *data was 12% for high and 8% for low NUTRIENT, which was within the targeted 15% range. The only parameter changed between the two conditions was *initialVal*, the level of NUTRIENT present outside the SMS during the simulation. For both conditions, CELLS used the same Table 3 set of operating principles. Under Methods, we describe that only minimal tuning of the indicated subset of the parameters in Table 2 was needed to achieve these matching growth characteristics.

### In silico doubling times were similar to *in vitro *doubling times

The measured doubling times for SMS and EMT6 spheroids were similar at high NUTRIENT levels, but quite different at low NUTRIENT levels, as shown in Table 4. At low nutrient levels, EMT6 cell number doubled every 17 hours, whereas the SMS required 40 HOURS. This apparent discrepancy was initially difficult to explain, considering that the growth curves were very similar. Some explanatory factors may include the high variability of the in silico results at small SMS sizes, as well as the experimental variability between *in vitro *trials. In addition, initial doubling times were calculated [13] using a best fit of the Gompertz equation [15] to data from multiple experiments. The Gompertz equation describes an exponential curve with an exponentially decreasing growth rate. It can be fit to many types of in vitro animal and tissue growth data. The equation used was $y=ae^{-e^{\left(b-ct\right)}}$, where *t *is time, *y *is cross-sectional area, and *a, b*, and *c*, are the parameters of the equation. When we fit the *in vitro *results (from the single experiment we used for validation) to the Gompertz equation, we obtained an initial doubling time of 21.6 hours at high nutrient levels and 26.4 hours at low, as compared to 19.2 HOURS for in silico simulations at high and low NUTRIENT, as shown in Table 4. These results are more consistent, which is not surprising, as we tuned *prolifDelay *and *proBias *to generate in silico doubling times that could be mapped approximately 1:1 to wet-lab doubling times. *ProlifDelay*, a stochastic parameter, is the average TIME interval that a CELL must wait before it has the option to create a new CELL. The value of *prolifCounter *specifies the interval for each CELL. Each *prolifCounter *value is calculated from *prolifDelay *as described under Methods. To enable a successful proliferation event, a pseudo-random number must exceed a specified value. The variable *proBias *specifies the probability distribution from which that value is drawn, as described under Methods. The larger the *proBias *value, the more extreme that distribution, making it increasingly difficult for a new CELL to be created.

**Table 4:** Comparison of *in vitro *and in silico growth characteristics.

**Condition**	**Initial doubling time**	**Viable rim width**	**Necrosis onset time**	**Necrosis onset size**	**Maximum area**
**In silico**					
High NUTRIENT*	21.4 HOURS/19.2 HOURS‡	245 μm	11.4 DAYS	0.253 mm^2^	1.61 mm^2 ^‡/1.46 mm^2^‡
Low NUTRIENT*	40.0 HOURS/19.2 HOURS‡	62 μm	4.4 DAYS	0.0266 mm^2^	0.0645 mm^2^‡
***In vitro***					
16.5 mM glucose & 0.28 mM oxygen	23.0 hours†/21.6 hours‡	240 μm	N/A	0.209 mm^2^	3.25 mm^2 ^†/2.79 mm^2^‡
0.8 mM glucose & 0.07 mM oxygen	17.0 hours†/26.4 hours‡	60 μm	N/A	0.0181 mm^2^	0.221 mm^2 ^†/0.0725 mm^2^‡

* Values for mean of ten runs.

† From Gompertz fit by Freyer and Sutherland [13].

‡ From Gompertz fit by authors.

### Measured viable rim widths were similar

Viable cell rim widths, eighth in Table 1, have been characterized, and were used to further validate SMS attributes. The data in Table 4 show that VIABLE SMS rim widths were close to *in vitro *values. Because the coefficient of variation of EMT6 spheroid areas was at least 29%, the corresponding value for radius was roughly 15%. We specified that any SMS radius within 15% of a referent radius would be acceptably similar because that radius would be experimentally indistinguishable from a repeat EMT6 experiment, had one been preformed. We observed a mean SMS rim width under the high NUTRIENT condition that mapped to 245 μm, compared to 240 μm *in vitro*, a difference of 2%. At low NUTRIENT, mean SMS rim width mapped to 62 μm, compared to 60 μm *in vitro*, a 3.3% difference. Although we performed some tuning of the critical levels required to remain in the PROLIFERATING or QUIESCENT state, these similarities are still noteworthy. They reinforce the likelihood that the principles of operation used by SMS CELLS may map to a corresponding set of operating principles used by EMT6 cells.

### NECROSIS onset and final saturation size were similar

Under high NUTRIENT, measures of SMS diameters at NECROSIS onset, listed in Table 4, achieved the targeted similarity measure. They were within 15% of those observed by Freyer and Sutherland [13]. Following SMS execution under high NUTRIENT, mean diameter at which the SMS first underwent NECROSIS mapped to roughly 530 μm, compared to 516 μm for the EMT6 spheroids under comparable conditions, a 2.7% difference. Under the low NUTRIENT condition, SMS underwent NECROSIS when the system reached a diameter corresponding to approximately 180 μm, compared to 152 μm for EMT6 spheroids, an 18.4% difference.

The maximum sizes attained by SMS were not similar to those predicted by Freyer and Sutherland [13], (Table 4). Freyer and Sutherland did not measure maximum sizes, but instead inferred them by fitting data to the Gompertz equation and then using the fitted equation to predict an expected maximum size. The data fit were averages of results from experiments on different batches of EMT6 spheroids. Because the SMS were being compared to data from a single experiment, we fit the Gompertz equation to that referent data (Fig. 3). Table 4 shows that the new result did not differ significantly from the one originally reported at high nutrient levels: the moderate in silico-*in vitro *discrepancy remained. However, the maximum fit size was smaller at low nutrient concentrations, down from 0.221 mm^2 ^to 0.0725 mm^2^. The maximum size reached by the SMS at low nutrient mapped to 0.0645 mm^2^, a difference of 11%. That was judged acceptably similar to our Gompertz equation fit. A reasonable conjecture for the discrepancy at high nutrient levels is that the set of operating principles used by cells in maturing EMT6 spheroids were somewhat different than the set used earlier, during spheroid expansion. Our goal was to seek one set of SMS operating principles that would enable validation for both high and low NUTRIENT conditions. It would be straightforward to relax that requirement and achieve improved similarity at high NUTRIENT levels.

### SMS shape and stability were controlled by *proBias*

**Figure 4:**
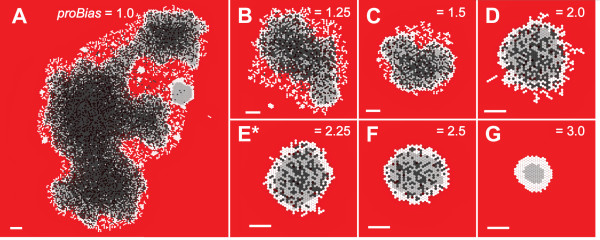
**SMS cross-sections at varied *proBias *values and low NUTRIENT**. All images were recorded at 18 DAYS. Scale bar: 100 μm. Other parameter values were as listed in Table 2. (A)-(G) *proBias *values are shown. *: *proBias *value in Table 2.

How important are the quantitative aspects of the AXIOMS in controlling spheroid shape and stability? AXIOMS 7 and 9 in Table 3 play critical roles in controlling SMS shape and stability. The consequences of their application in concert with the other seven depend to a large degree on the value assigned to the parameter *proBias*, which, as explained under Methods, influences the likelihood of stressed CELLS to proliferate. We conducted experiments at varied levels of *proBias*. Results are shown in Fig. 4. Increasing *proBias *improved an SMS's ability to fill in fissures that formed after growth stabilization. Low levels of *proBias *enabled fissures to reach toward the SMS center, destabilizing the structure and causing chaotic, uncontrolled growth. We have found no evidence that this destabilizing mechanism maps to *in vitro *counterparts. However, it may demonstrate a principle: surface irregularities can affect a spheroid's growth rate. An SMS that has elongated to form a rod-like structure could not easily increase its width because of limited NUTRIENT availability. However, absent other constraints, nothing would prevent it from elongating further. Clearly, there are other factors and forces involved in maintaining the stability and shape of large spheroids *in vitro*. However, they are beyond the SMS's current scope. SMS are capable of maintaining stable shapes for a specified TIME within certain parameter ranges, outside of which new AXIOMS and/or non-local constraints would be required.

### SMS long-term shape changes lead to instability

**Figure 5:**
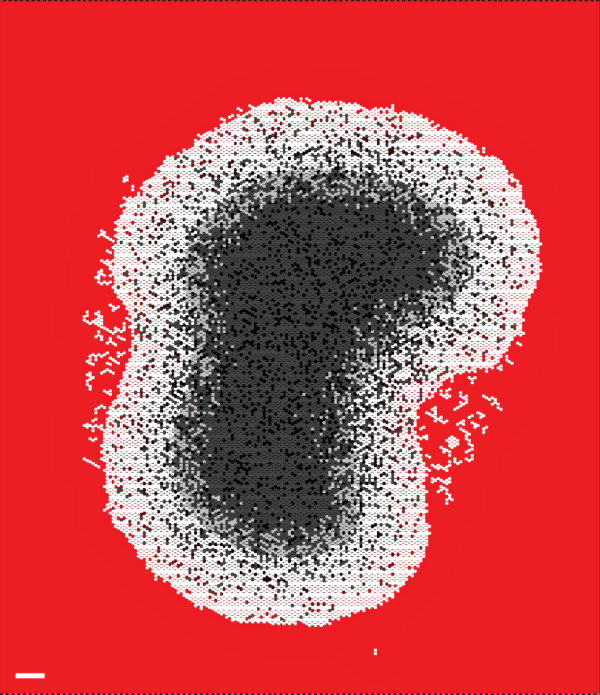
**An SMS cross-section at 67 DAYS at high NUTRIENT level**. SMS shape is no longer circular. Scale bar 100 μm. Parameter values were those listed in Table 2.

The AXIOMS used to manage CELL STRESS were effective at maintaining stable small and medium sized SMS. However, when growth was extended beyond 50 DAYS under high NUTRIENT, SMS began to lose their circular shapes. The 67-DAY old SMS in Fig. 5 is an example. Because of the stochastic nature of the events involving each CELL, the growth trajectories, shapes, and sizes of separate SMS executions can be different under identical conditions. For the same reason, small regional differences in large, mature SMS can gradually become amplified, resulting in large subregions having measurably different characteristics. This leads to an unstable system. Measures of SMS long-term growth at high and low NUTRIENT are provided in Additional file 1, Fig. S1. EMT6 spheroids do exhibit varied shapes during growth. However, we are not aware of *in vitro *observations that can be used to validate this SMS behavior, possibly because it is challenging (and expensive) to maintain large EMT6 spheroids in culture for 50 days or more (although Chignola et al. have maintained Rat 9L spheroids to 70 days [16]). If maintenance of generally circular shapes beyond 50 days under high NUTRIENT were to be added to the targeted attributes list, the current SMS would be falsified. Inclusion of additional mechanisms would be needed to reestablish validation.

### Varying parameters changed growth curve and SMS shape

**Figure 6:**
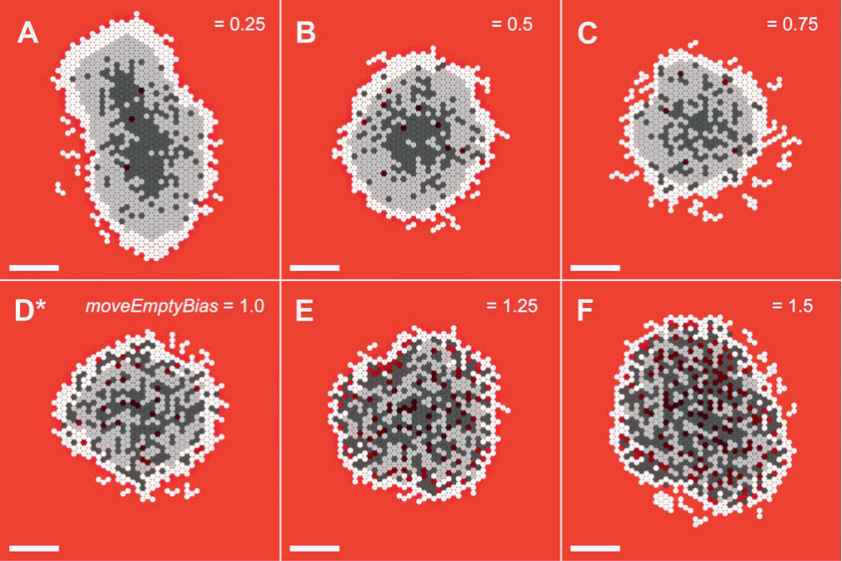
**SMS cross-sections at varied *moveEmptyBias *values and low NUTRIENT**. All images were recorded at 21 DAYS. Scale bar: 100 μm. Other parameter values were as listed in Table 2. (A)-(F) *moveEmptyBias *values are shown. *: *moveEmptyBias *value in Table 2. Cross-sections at *moveEmptyBias *= 0 are not shown because they grew too quickly and filled the available space before 21 DAYS elapsed. As *moveEmptyBias *increased, more empty spaces were visible within the SMS.

**Figure 7:**
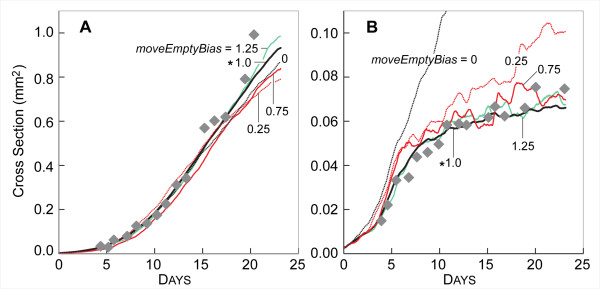
**Influence of *moveEmptyBias *on SMS growth**. Gray diamonds: *in vitro *data as in Fig. 3. Other parameter values were those listed in Table 2. Colored lines are results of single experiments for the indicated values of *moveEmptyBias *from 0 to 1.5 (*moveEmptyBias *= 0 plus the same values as in Fig. 6). (A) high NUTRIENT; (B) low NUTRIENT. *: *moveEmptyBias *value in Table 2.

We conducted experiments in which we varied parameter values and observed the effect on measures of SMS growth and morphology. The results, summarized in Table 5, indicate whether increasing a parameter increased, decreased, or did not affect a specific measure (such as maximum size reached). In addition, we examined the consequences of changing parameter values in more detail. Changing *moveEmptyBias *had a limited but significant effect on SMS morphology (Fig. 6) and growth (Fig. 7). The parameter *moveEmptyBias *influenced movement of CELLS exposed to the outside surface of the SMS or adjacent to a fissure. The larger the value of *moveEmptyBias*, the less likely a CELL at the edge experiencing low STRESS (defined under Methods) would move into an adjacent empty space when given the opportunity. A larger *moveEmptyBias *value hindered fissure elongation. *MoveEmptyBias *was tuned empirically to control SMS shape but still allow CELL-free spaces to exit the SMS rather than be trapped inside for an extended duration. CELLS with larger *moveEmptyBias *values experiencing low STRESS rarely moved into adjacent empty spaces, whereas CELLS under high STRESS are likely to do so.

**Table 5:** Effects of increasing parameters on in silico measures.

**Parameter**	**Maximum size**	**Viable rim width**	**Quiescence onset**	**Necrosis onset**	**Growth rate**	**Doubling time**	**Stability**	**Necrotic core size**
*DiffusionRate*	↑	↑	↑	↑	--	--	↔	↔
*initialVal*	↑	↑	↑	↑	↓	↓	↔	↑
*proConsumeRate*	↓	↓	↓	↓	--	--	--	↔
*quiConsumeRate*	↓	↓	--	↔	--	--	--	↔
*prolifDelay*	↔	--	↑	↑	↓	↓	--	↔
*removeDelay*	↑	--	--	--	--	--	↔	↑
*proNut*	↔/--	--	↓	↓	--	--	↓	↔
*quiNut*	↓	↓	--	↓	↓	--	--	--/↓
*proBias*	↓	↑	↑	↑	↓	↓	--/↑	↓
*moveEmptyBias*	↔	↓/↑	--	--	↑	--	↑	↑

↑: Positive effect. ↓: Negative effect. ↔: Effect limited or not easily determined. --: No effect. Cells with two values (e.g. --/↑) indicate different effects at high nutrient (left) and low nutrient (right). Cells with one value indicate similar effect at high and low nutrient.

* Quiescence onset and necrosis onset are the measured times of onset. A positive effect indicates that the time of quiescence or necrosis onset increased, thus they occurred later in the simulation.

As demonstrated by Fig. 7, when *moveEmptyBias *was set to zero, SMS grew linearly at a high rate in low NUTRIENT conditions and failed to saturate. Fissures appeared, which caused the SMS to destabilize and grow chaotically. Increasing *moveEmptyBias *by as little as 0.25 resulted in almost complete SMS saturation. Further increasing *moveEmptyBias *did not significantly affect growth rates or stability, but small changes were evident at both low and high NUTRIENT levels. While *moveEmptyBias *does not directly map to an *in vitro *quantity, these results indicate that there may be threshold values for shape maintenance mechanisms, below which an EMT6 spheroid would generally become unstable.

**Figure 8:**
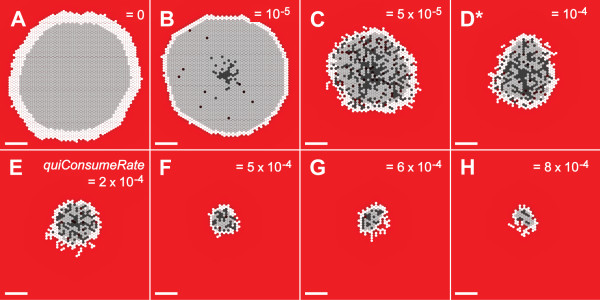
**SMS cross-sections at varied *quiConsumeRate *and low NUTRIENT**. All images were recorded at 13 DAYS. Scale bar: 100 μm. Except for *quiConsumeRate*, parameter values were those listed in Table 2. (A)-(H) *quiConsumeRate *values are shown. *: *quiConsumeRate *value in Table 2.

**Figure 9:**
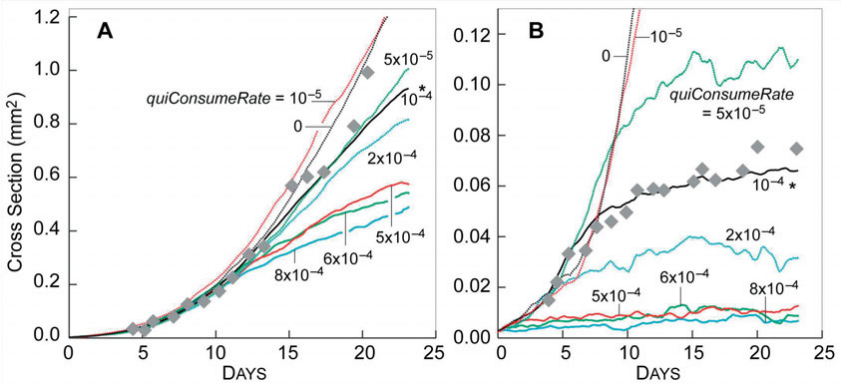
**Influence of *quiConsumeRate *on SMS growth**. Gray diamonds: *in vitro *data as in Fig. 3. Other parameter values were those listed in Table 2. Colored lines are results of single experiments for the indicated values of *quiConsumeRate *from 0 to 8.0 × 10^-4 ^(same values as in Fig. 8). (A) high NUTRIENT; (B) low NUTRIENT. *: *quiConsumeRate *value in Table 2.

The value of *quiConsumeRate *determined the amount of NUTRIENT per SECOND consumed by each quiescent CELL. The value was tuned between zero and *proConsumeRate*. Varying *quiConsumeRate *produced consistent and dramatic results. Increasing the amount of NUTRIENT consumed by QUIESCENT CELLS reduced the number of QUIESCENT CELLS capable of existing within the SMS. The consequences are clearly visible in Fig. 8: as *quiConsumeRate *increased, the width of the viable rim decreased, as did the number of QUIESCENT CELLS in the system and overall SMS size. Figure 9 shows that the growth rate and saturation size steadily decreased. *ProConsumeRate *specified the amount of NUTRIENT per SECOND consumed by proliferating CELLS. For simplicity, PROLIFERATING CELLS consumed the same amount of NUTRIENT regardless of whether they were actively creating a new CELL, waiting for an opportunity to do so, or were unable to do so because they were surrounded by other CELLS. *ProConsumeRate's *value in Table 2 was purposefully selected to be within the range of glucose consumption rates reported in [17].

**Figure 10:**
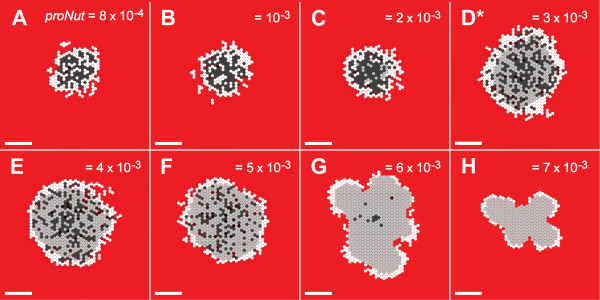
**SMS cross-sections at varied *proNut *and low NUTRIENT**. All images were recorded at 18 DAYS. Scale bar: 100 μm. Other parameter values were those listed in Table 2. (A)-(H)*proNut *values are shown. *:*proNut *value in Table 2. Note that while size increased initially with increasing *proNut *(a consequence of increased numbers of lower-consumption rate quiescent CELLS), larger *proNut *values caused the proliferating rim to become so thin that the SMS destabilized. The more quickly CELLS that are incapable of proliferating transition to the (lower-consumption) QUIESCENT state, the larger the size of the stable SMS.

**Figure 11:**
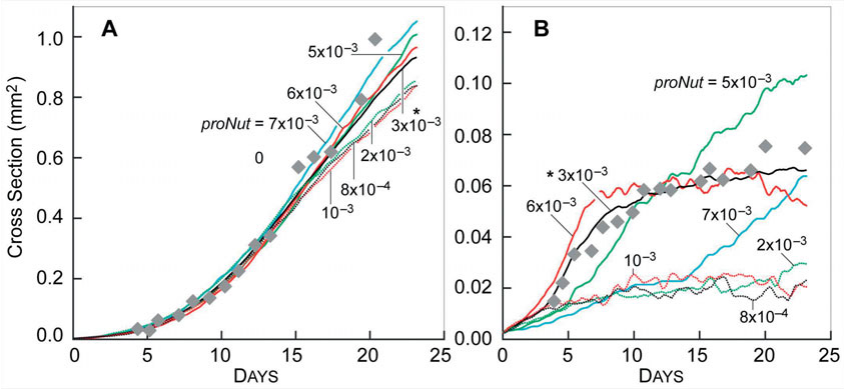
**Influence of *proNut *on SMS growth**. Gray diamonds: *in vitro *data as in Fig. 3. Other parameter values were those listed in Table 2. Colored lines are results of single experiments for the indicated values of *proNut *from 8.0 × 10^-4 ^to 7.0 × 10^-3 ^(same values as in Fig. 10). (A) high NUTRIENT; (B) low NUTRIENT. *: *proNut *value in Table 2.

A CELL switched from PROLIFERATING to QUIESCENT state when the amount of NUTRIENT at its location dropped below the value of the parameter *proNut*. If NUTRIENT later increased above *proNut*, the CELL returned to the PROLIFERATING state. Changing the value of *proNut *changed the amount of NUTRIENT that CELLS required to remain in the PROLIFERATING state. When set to 8.0 × 10^-4^, CELLS transitioned directly from the PROLIFERATING to the NECROTIC state, as shown in Fig. 10. We do not distinguish between simulated necrotic and apoptotic cell death, instead conflating both into removal of NECROTIC CELLS. When referring to *in vitro *research we defer to the original documents for terminology. CELL growth at that setting under low NUTRIENT (Fig. 11) was low, as PROLIFERATING CELLS consume more NUTRIENT than QUIESCENT CELLS. Increasing *proNut *to 2.0 × 10^-3 ^produced little change in morphology or growth rate, but both measures changed dramatically when *proNut *was raised to 3.0 × 10^-3^. At that value, a population of QUIESCENT cells became clearly evident, and the growth rate and stable maximum size was noticeably larger. That trend did not continue, however. As *proNut *increased further, first to 4.0 × 10^-3 ^and then to 5.0 × 10^-3^, only small changes in morphology and growth curves were evident. The population of QUIESCENT CELLS was only slightly larger. Another sharp change was evident as *proNut *reached 6.0 × 10^-3^: the SMS destabilized (Fig. 10G) and the growth curve did not plateau (Fig. 11B). The results suggested that a window existed within which the number of PROLIFERATING CELLS, having higher consumption rates, balanced the number of QUIESCENT CELLS, which had lower consumption rates. When the level of NUTRIENT within a location dropped below the value *quiNut*, the CELL switched irreversibly to the NECROTIC state. As is evident from Additional file 1, Figs. S2 and S3, varying *quiNut *had less complex effects. As *quiNut *was increased, the growth rate, saturation size, and viable rim width all decreased.

**Figure 12:**
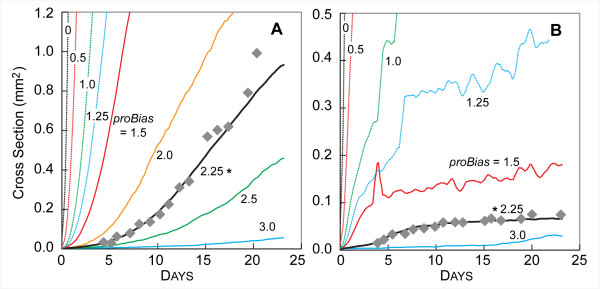
**Influence of *proBias *on SMS growth**. Gray diamonds: *in vitro *data as in Fig. 3. Other parameter values were those listed in Table 2. Colored lines are results of single experiments for the indicated values of *proBias *from 0 to 3. (A) high NUTRIENT; (B) low NUTRIENT. *: *proBias *value in Table 2.

The consequences of changing *proBias *at low NUTRIENT were potentially confusing, because there were two dramatic and different effects on SMS growth and morphology. The first effect, clearly observable in Fig. 12B, was an increased growth rate when *proBias *was decreased from the default validation value of 2.25. CELLS that used smaller *proBias *values were able to create new CELLS even when their stress was higher. As a result, they created new CELLS more frequently, increasing the overall growth rates. The increase in growth rate confounded our ability to analyze the changing stability of the SMS, which was the second major effect of changing *proBias*. At very low values of *proBias*, individual CELLS proliferated quickly, and the SMS grew to the edge of the space within a few DAYS (images not shown). Consequently, it was not possible to determine whether the SMS was more or less stable at these values without adjusting other parameters, such as *prolifDelay*. At values of 1.0 and 1.25, the growth rate decreased, and the SMS was relatively unstable. As *proBias *increased further (1.5, 2.0, 2.25), the SMS became stable once again, though the growth rate decreased as the value was raised further (2.5, 3.0). We believe that if *prolifDelay *were adjusted in concert with *proBias*, maintaining the same initial doubling rate, the SMS would become unstable at low values of *proBias*. At high NUTRIENT levels, shown in Fig. 12, the SMS growth rate decreased as *proBias *increased, but the relative SMS stability did not change.

## Discussion

SMS events and mechanisms were not intended to be exact replicas of the actual physical or chemical events ongoing *in vitro *during EMT6 spheroid growth. Nor were predictions of specific events part of the intended SMS use. Rather, the intent behind our method has been, given a set of EMT6 spheroid attributes, to discover SMS computational mechanisms that might map logically and intuitively to in vitro counterparts. This has been accomplished by exploring the inverse map from phenomena to mechanism. The primary functional unit of an SMS – a CELL – does map 1:1 to an EMT6 cell. Because an EMT6 cell is autonomous, we designed SMS CELLS to be quasi-autonomous. SMS CELLS currently have no internal components. As atomic software objects, they needed operating principles to function. Most of the principles that cause an EMT6 cell to act in a particular way when faced with specific circumstances in culture were unknown. Consequently, we needed to discover and implement operating principles that each SMS would use, evaluate those mechanisms through simulation and observation, and modify them based on the results. Following [5,7], CELL operating principles were formulated as AXIOMS. Their specifications were tightly guided by available knowledge of EMT6 behaviors in culture [17-20]. By iteratively following the diagram in Fig. 1, we narrowed and refined early candidate AXIOMS to nine. These AXIOMS were refined further so that measures of SMS growth characteristics would match prespecified, iteratively expanded, targeted sets of EMT6 spheroid growth characteristics according to specific similarity measures. Having achieved that objective, we suggest that the resulting SMS operating principles (Table 3) can stand as an abstract representation of EMT6 operating principles under comparable growth conditions. We posit that the larger the targeted set of EMT6 attributes satisfactorily matched, the more realistic the mapping between SMS and EMT6 operating principles.

It is significant that within a simulation cycle one CELL can apply more than one AXIOM. This reflects the complexity inherent in even the simplest interpretation of a biological system. The amount of nutrients or growth factors in the environment, for example, can be independent of whether a cell is surrounded by other cells or isolated.

Use of AXIOM 8 in combination with the others resulted in an extreme degree of contact inhibition: CELLS that were surrounded by other CELLS did not create new CELLS. That was a purposeful simplification. Nevertheless, the targeted attributes were achieved. The evidence indicates that some cell proliferation does occur throughout EMT6 spheroids [14], but that the frequency decreases dramatically with distance from the surface. If those observations were to be added to the list of targeted attributes, it would falsify the current SMS. Validation against that expanded attribute set would require increasing SMS complexity, possibly revising, as well as extending the list of AXIOMS. Relative to the current SMS, the fraction of CELL creation events occurring at the surface would be reduced and counterbalanced by division events occurring elsewhere. Because SMS components are quasi-autonomous, when the current set of targeted attributes is expanded one at a time, it is relatively straightforward to revise an SMS to match each new, expanded set. We achieved the targeted attributes using an SMS CELL that exists in three states. When the attribute list is expanded (even to include pathological attributes of drug treatments), it is straightforward to add new CELL states that possess different axiomatic operating principles.

The current set of abstract, axiomatic operating principles is believed to be the source of the discrepancy between in silico and *in vitro *growth at high NUTRIENT (Fig. 3). The SMS can be parameterized so that simulated growth more closely matches the higher NUTRIENT data (not shown), but at the expense of achieving a much poorer match to the low NUTRIENT data. Note that the differences in growth properties at low and high concentrations of oxygen and glucose are more extreme for the referent data than is seen with other available sets of growth data, such as the data used by [2]. Achieving a tighter match would require adding more detail.

Whereas Freyer and Sutherland described the inhibitory actions of a tumor extract on proliferating cells [19], they did not separate the components to identify the source of inhibition. LaRue et al. [14] observed cyclin-dependent kinase inhibitors that are associated with cell-cycle arrest, but they did not demonstrate a causative role. Researchers have speculated that a factor in spheroid growth stabilization may be cell inhibition caused by some material being released from necrotic cells [14,19]. We did not include such an attribute among those targeted, in part because it had not been confirmed. Nevertheless, the current SMS successfully produced stable spheroids without the production and action of such a factor, effectively establishing that one is not required for growth stabilization at biologically realistic SMS parameter settings. Of course, we cannot conclude from this in silico evidence that a necrotic inhibitor is absent *in vitro*. It is instead evidence that EMT6 spheroid growth stabilization need not *require *the presence of such an inhibitor. It is also useful to contrast the modeling approach used here with that used by [21,22]. Longo et al., having achieved some degree of satisfaction about the mechanisms implemented, focused on replication and prediction of referent results from particular AXIOMS in an exploration of the forward map from generator to phenomenon. Our approach focused on discovering appropriate AXIOMS, such as the need for a potential inhibitor or a particular arrangement of neighboring components, and which were necessary and/or sufficient. We relied on falsification to select from the plausible generators.

CELLS that experienced a high STRESS were likely to move to reduce STRESS, while CELLS experiencing low STRESS were likely to proliferate and create more CELLS. As shown in Fig. 5, some large SMS destabilized during long-term growth. We determined that this behavior was caused by the probabilistic, local nature of the STRESS based movement and proliferation algorithms. At small SMS sizes, all deviations from the minimum-STRESS, convex curvatures are corrected by the movement and proliferation algorithms within a small number of simulation cycles. For much larger sizes, however, local curvature can be within the variability of the STRESS algorithm, yet the shape that emerges can be non-circular and irregular. That is because all AXIOM preconditions used only local information. When SMS are very large, the surface adjacent to every surface CELL can be relatively flat (the CELLS are experiencing low STRESS), yet the overall SMS can be non-circular. If needed, the effect could be minimized in several ways, all of which would require increasing SMS complexity. The simplest for the current SMS design would be to enable sharing information about each CELL'S current STRESS with a larger cluster of neighbors.

Although other models have not explicitly controlled spheroid shape, they have nevertheless done so implicitly. For example, by placing an adhesion term in their models, Schaller et al. and Jiang et al. caused CELLS to cling together, thus minimizing surface irregularities [2,6]. In fact, Schallar et al. noticed differences in overall shape when they used different values for the adhesion parameter. Anderson et al. found that changing the EXTRACELLULAR MATRIX structure in a simulated model of tumor invasion produced dramatic differences in tumor morphology [23]. Our analogue did not initially contain a mechanism to control SMS shape, but we found that the analogue could not mimic the targeted attributes without one. Although the SMS did not explicitly define and implement cellular adhesion like [2,6], stress based movement and proliferation produced a similar effect.

## Conclusion

We presented an idea: under the conditions of EMT6 spheroid growth in culture, molecular cell biology manifests at the cell level in what can described as a small set of operating principles that are responsible for the characteristic *in vitro *phenotypic attributes. We anticipated needing to identify and understand the operating principles in order to better understand how specific, detailed subcellular events may be linked to attributes of systemic EMT6 spheroid growth. Our method and approach are diagrammed in Fig. 1. We designed, refined, and tuned quasi-autonomous software components that, upon execution, formed abstract SMS analogues. We showed that measures of SMS behaviors during simulated growth were similar to available wet-lab data using a quantitative similarity measure. We submit that SMS mechanisms, with emphasis on the explicit AXIOMS, may stand as a plausible, abstract hypothesis for what was observed during those EMT6 spheroid growth experiments.

A future challenge will be to build a parallel system in which each (or some) atomic CELL component and its operating principles are replaced with a composite CELL object containing a set of interacting components intended to map to modular components within EMT6 cells. During INTRACELLULAR interaction, specific internal components would each use a portion of the same local environment information to act on other internal components such that actions are essentially identical to the current SMS events. The resulting growth characteristics would be indistinguishable from those described herein. The two systems could be iteratively advanced in parallel as new information and data were added to the set of targeted attributes. Using cross-model validation in that way is expected to provide a systematic strategy to answer the third of the three questions posed in the Introduction. What organization of modular and molecular biological details enables operating principles to emerge, and be sustained at the cell level?

## Methods

### *In vitro *system: historical context

Because cancer is such a complex and heterogeneous disease, researchers develop and study model systems. One is the *in vitro *EMT6/Ro multicellular tumor spheroid system. Freyer and Sutherland used the system to study avascular cancers in the 1970s and 1980s [13,19,24-27]. Their initial goal was to create a system that would allow many EMT6 spheroids to be grown in the same flask under identical, controlled conditions. Study of that model was expected to improve our understanding of how early stage cancer forms and improve our ability to treat it when the cells have not reached total genomic instability and still have much in common with normal cells [28].

In order to obtain adequate numbers of spheroids for measuring growth curves, experiments employed spinner flasks containing hundreds of spheroids. The cultures were initiated in monolayer and then grown in dishes until small spheroids were present (95–100 hours). These spheroids (usually 400–600 cells) were sorted and transferred to flasks, which contained a solution of glucose-free Eagle's Basal Medium, Fetal Bovine Serum, and varied concentrations of glucose. Oxygen was bubbled through the flask, and glucose was replenished roughly every 10–14 hours [13]. Most early experiments were designed to characterize the system and its behavior. Eventually, however, many researchers shifted to using the system as a tool rather than studying the system itself. The seminal studies describing the behavior and characteristics of EMT6 spheroids were primarily completed by 1992. Important work continued nevertheless. There was an effort [14] to identify a potential necrotic inhibitor, and confocal microscopy was used to assess growth fractions [29].

### Targeted attributes describe *in vitro *EMT6 spheroid growth

Although there is variation in EMT6 spheroids' growth, it reliably follows the same well-defined pattern [24]. EMT6 spheroids initially grow exponentially without constraint from nutrient or other cells. This gives way to linear growth as cells become quiescent due to nutrient depletion within the spheroids. Eventually the spheroids begin stabilizing, both in volume and cell number, though cells continue to reproduce on the outer edge. They develop a concentric layered structure: an outer layer of actively proliferating cells, a middle layer of quiescent cells, and a core of necrotic cells and cellular debris. The material released by dying cells is thought to inhibit cellular proliferation [14], but it is not clear if this material actually affects cells in the rim and thus spheroid growth rates. During growth, an EMT6 spheroid maintains a generally spherical shape, but neither shape nor relative EMT6 spheroid stability have been quantified, which makes shape validation difficult. Additionally, though the width of the viable rim has been measured for different cell types, only one group has attempted to quantify the ratio of proliferating and quiescent cells [29]. Wartenburg and Acker performed these measurements on human glioma spheroids. They do form spheroids with concentric layers, but have quantitative growth characteristics (such as initial doubling time and saturation size) that are different from those of EMT6 spheroids. We elected to falsify and validate our experimental results using data from Freyer and Sutherland [13], because they performed the most thorough and complete analysis of EMT6 spheroid growth, including measuring growth curves, the width of the viable rim, and the size at the approximate time of necrosis onset.

### Analogue construction within an agent-based paradigm

SMS construction used agent-based methods available in the simulation toolkit MASON [30]. This framework was used for data generation, scheduling, and visualization. The SMS is an example of a class of simulation models referred to as executable biology [31,32]. Our simulation shares some similarities with [21] and is closely related to [33], though the system under study and the simulation framework are distinct. Executable biological analogues are poorly suited for precise prediction, but are ideally suited for testing hypotheses about mechanisms. The basic method requires building mechanisms at the functional unit level closest to the targeted phenomena. Here, that unit is the cell. An SMS is comprised of quasi-autonomous agents. Each maps to an EMT6 cell. The initial limit for SMS resolution was the CELL. If achieving the list of targeted attributes required doing so, the resolution of the SMS could be increased. CELLS interact with each other and their environment during each simulation cycle within a two-dimensional, hexagonal grid. Earlier versions used a square grid, but in addition to requiring a higher order implementation of discrete diffusion, it also generated artifacts that are not present with the hexagonal grid. We tested different orders of discrete diffusion to verify that artifacts were not caused by the diffusion algorithm. CELL actions are mandated by AXIOMS[5,7]: when a specified condition is met, a specified action occurs. Together, these AXIOMS are a CELL's operating principles. A goal has been to improve the variety of SMS attributes that are similar to corresponding EMT6 spheroid attributes: the expectation being that with increasing phenotypic similarity, the higher the likelihood SMS AXIOMS will map to corresponding EMT6 operating principles (Fig. 1). As the list of targeted attributes expands, analogue resolution can be adjusted as needed.

A second grid, adjacent to the CELL'S grid contains a diffusible substance called NUTRIENT. NUTRIENT adjacent to each CELL is detected by and available to that CELL for consumption. During each simulation cycle, each CELL uses the AXIOMS in Table 3 to select actions based on how its local environment has changed since the last simulation cycle. Examples of actions include move, change state, create new CELL, DIE, and shed. AXIOMS are implemented by algorithms that utilize the parameter values listed in Table 2. Where appropriate, parameter values intentionally mirror values measured *in vitro*. The remaining parameters were tuned using an iterative process: change parameter value, execute, evaluate relative to referent observables, cogitate, change again, etc.

### Tuning parameter values improves the analogue's ability to survive falsification

Once a set of targeted attributes had been specified, parameter tuning and SMS validation became closely linked. When seeking fundamental necessary and sufficient in silico mechanisms, we incremented the complexity upwards. We started with the simplest possible system and used an iterative falsification process, beginning with the first of the targeted attributes listed in Table 1. That iterative refinement method, of which parsimony is a factor, has been used successfully in addressing other simulation goals [34]. While exploring early AXIOM specifications and the in silico conditions needed to achieve the first attribute, we mostly ignored our larger knowledge of EMT6 spheroid biology. At that stage, the analogue had one and only one goal: achieve the targeted attribute. Once that was achieved, that early SMS was valid *for that one targeted attribute*. As shown in Table 3, during the process of achieving Attributes 1 and 2 individual AXIOMS were qualitatively validated against their *in vitro *counterparts. For instance, we verified that individual CELLS did not create new CELLS more frequently than is observed *in vitro*. In this context, an analogue was considered valid if it exhibited attributes that matched the targeted set according to some prespecified similarity measure. We then added a new attribute, such as no. 2 in Table 1, to the targeted list. Doing so often (but not always) immediately falsified that SMS, which was the case with the addition of attribute no. 2. To revise the construct and form a new, more valid SMS with the expanded set of targeted attributes, we found it essential to introduce a volume loss mechanism and a mechanism to stabilize SMS growth and shape: we added CELL shedding and STRESS states along with AXIOMS to manage those new states. The new AXIOMS necessitated adding new parameters: *moveEmptyBias and proBias*. Following a period of iterative refinement, these additional mechanisms enabled the SMS to survive our attempts to falsify it with the expanded attribute list. We executed that same protocol for each of the other attributes in Table 1. Following each expansion of the attribute list, we reconsidered all AXIOMS, revising and merging parsimoniously where needed. We initially coarse-tuned parameter values and subsequently fine-tuned them. We continued that process for all attributes listed in Table 1, until the SMS was able to produce the matching growth curves in Fig. 3. That same iterative refinement method can be used to further improve SMS behaviors, and – presumably – bring SMS principles of operation into closer alignment with those of EMT6 cells. Our explicit process of iterative falsification contrasts to most prior work in executable biology, including [21,33], which describe the completed models and predictions, but do not list the attributes targeted for reproduction or the order in which they were achieved. We believe added transparency will allow others to build on the work described here.

Some in silico parameters, such as the *diameter *of a CELL, mapped directly to measured observations of *in vitro *EMT6 quantities. These are noted by their source in literature within Table 2. One exception was the mean value of the in silico interval between when a CELL entered the NECROTIC state and when it was removed (creating an empty space). A value of five HOURS (18,000 SECONDS) was used. Doing so required three assumptions. The first was that the experimental setup used by Harris et al. to obtain these measurements did not contribute excessively to the measured apoptosis duration [35]. The second was acceptance of the authors' assumptions about apoptosis: apoptosis begins when apoptotic morphology was observed and ended when the cell began to fragment. The final and most significant was that we could map these values to EMT6 cells undergoing necrotic cell death induced by nutrient depletion.

The actual number of simulation cycles that elapsed from when a CELL became NECROTIC and when it disappeared depended on the value of *removeCounter*, a pseudo-random number (PRN) drawn from a uniform distribution over the interval [0-*removeDelay*). *RemoveCounter *was decremented each cycle that a CELL was in the NECROTIC state, resulting in its removal when the value reached 0. Setting *removeDelay *to 36,000 SECONDS (SEC), resulted in a mean *removeCounter *= 18,000 SEC, which mapped directly to the reported mean duration of apoptosis [35].

In order to achieve the targeted attributes, it was sometimes necessary to select parameters that mapped to values that were toward the extreme end of an observed, referent range. For instance, in order to avoid excessive NUTRIENT consumption resulting in premature appearance of NECROSIS, CELLS consumed NUTRIENT at a rate of 5.0 × 10^-17 ^MOL/CELL/SEC. Observed glucose consumption rates were between 5.5 × 10^-17 ^and 36.0 × 10^-17 ^mol/cell/s [17].

Once a subset of parameter values had been set to map to *in vitro *counterparts, the remaining parameter values were tuned empirically so that the similarity between SMS and *in vitro *attributes achieved a specified measure of similarity. Previous agent-based simulation projects demonstrated that the empirical tuning approach is an effective strategy for locating biologically relevant regions of an analogue's parameter space [5,7,35]. Initially, parameter values were varied extensively to discover ranges for which qualitative SMS behavior could be mapped to a corresponding biologically plausible behavior. For instance, if *prolifNut *(the value that must be exceeded for a CELL to remain in the PROLIFERATING state) was higher than *initialVal*, proliferation did not occur. Similarly, we found that *moveEmptyBias *had to be higher than 0.5 to prevent fissure formation and eventual SMS destabilization. Following empirical tuning to the *in vitro *doubling times of 18 to 24 hours [13], we selected a value of 2.25 for *proBias *and 800 SEC for *prolifDelay*. Each CELL had its own individual *prolifCounter *that specified the number of SEC that must pass before it attempted to create a new CELL. We calculated *prolifCounter *using the method in Walker et al. [36]: *prolifCounter *= *prolifDelay*/2 + R_G_, where R_G _was a pseudo-random number drawn from a Gaussian distribution having mean = *prolifDelay*/2 and standard deviation = *prolifDelay*/10 [36]. Consequently, the average *prolifCounter *value was roughly equal to *prolifDelay*. Once parameter ranges were identified that achieved the targeted measure of similarity, each parameter was adjusted in sequence over a narrow range, and the consequences for SMS properties were recorded. Values that brought simulated behaviors closer to targeted values were retained. The parameter values obtained following that protocol are identified in Table 2. Note that with the possible exception of the critical NUTRIENT levels, none of these tuned parameter values map directly to measurable *in vitro *counterparts, and it would be problematic to obtain such values through experimentation.

### Measuring in silico and *in vitro *values

*In vitro *doubling time is the time required for an average EMT6 cluster to grow from 600 to 1,200 cells [13]. These numbers corresponded to an SMS expanding from 8.6 × 10^-3 ^mm^2 ^to 1.35 × 10^-2 ^mm^2^, which we used to determine SMS doubling time. Both high and low NUTRIENT VIABLE rim values were calculated by averaging the VIABLE rim width at NECROSIS onset and at the end of the simulation. Individual values of VIABLE rim width were found by counting the number of CELLS between the SMS center and the edge in three directions: from right of center, above the center, and diagonally left of the center. These three values were averaged to obtain the final width. Because the initial occurrence of NECROSIS was not necessarily stable (during early growth NECROSIS could appear and later vanish), we estimated the time of NECROSIS onset by moving backward; we specified it to be the latest time at which no NECROTIC CELLS were present. The method is similar to the one used by wet-lab researchers to estimate necrosis onset [13]. As done with EMT6 spheroids, the maximum SMS size reached was estimated by fitting the Gompertz equation to growth data and taking the maximum size predicted by the equation.

Growth rates *in vitro *were based on mean measures of spheroid diameter [13]. We converted those values into cross-sectional area in order to compare them with our in silico results. To establish a measure of SMS cross-sectional area we followed the method used *in vitro*, adding to the explicit phenomenological mapping between the SMS and its referent. We assumed SMS are roughly circular, which our observations demonstrated was the case. We first calculated the X and Y extents defined as follows: largest X (East-West or left-right) and Y (North-South or up-down) differences between CELLS at the edge of the SMS, ignoring detached, isolated CELLS. Those two values were averaged to obtain the measure of SMS diameter used to calculate area. This measurement adequately described the trends in SMS growth and remained quite close to the actual area occupied by all CELLS.

### Analogue environment on a hexagonal grid

The width of each grid location mapped to 10 μm. Each location was either empty or held a single CELL. A second, identical sized hexagonal grid was overlaid on the first. It contained the NUTRIENT consumed by CELLS, and its value was specified using a floating-point value from 0 to 1. The NUTRIENT within the system mapped primarily to glucose, but other medium components, such as diffusible growth factors, were conflated into the referent. At this early stage, the targeted attributes selected did not include a requirement for glucose and oxygen being separate components. The single NUTRIENT factor was deemed sufficient. NUTRIENT diffusion used a discretization of the two-dimensional continuous diffusion equation *du/dt = D*∇^2^*u*, where *D *is the diffusion constant and *u *is the amount of diffusible material. NUTRIENT moved from high to low density areas. In a given simulation cycle, each location in NUTRIENT space calculated a new value based on the values of itself and its neighbors during the previous cycle using a method adapted for the hexagonal grid from [37]. The new value was *u*_*new *_= *u *(1 - λ) + λ (*u*_*NE *_+ *u*_*SE *_+ *u*_*S *_+ *u*_*SW *_+ *u*_*NW *_+ *u*_*N*_)/6, where λ is the discretized diffusion value and *u*_*NE*_, *u*_*SE*_, etc. are the NUTRIENT values at the neighboring locations.

CELLULAR actions, such as NUTRIENT consumption and creating new CELLS (proliferation), occur on larger timescales than diffusion, so for convenience, the NUTRIENT space underwent ten steps for each SEC in CELL space. Having multiple time scales allowed for model accuracy without wasting computation time. Physical and temporal resolutions were purposefully mapped to specific values obtained from the EMT6 system in order to improve simulation realism and help ensure that observed SMS behaviors were not artifacts of unrealistic scaling. The desired time step (Δt) for diffusion within the analogue was related to the unit distance (Δx), *D*, and λ, such that Δt = 3Δx^2^λ/8*D *[38]. For the purposes of the simulation, λ = 0.28 was chosen in order to allow a Δt of 0.1 SEC when Δx is 10 μm and *D *is 105 μm^2^/SEC[17].

The NUTRIENT space was replenished by an algorithm that detects which empty locations lie outside the SMS and which lie inside. Empty locations outside the SMS were replenished to *initialVal *every ten SEC. We replenished during a simulation more frequently than was done *in vitro *in order to simulate the effect of stirring within EMT6 cultures: diffusion is not responsible for moving glucose and oxygen toward the EMT6 spheroid during growth, only through it [17]. In order to calculate which depleted spaces resided inside and which were outside, the replenishment algorithm completes multiple passes from the top left corner of the grid to the bottom right and back. On each pass any empty space that is adjacent to an outside empty space is labeled as outside the SMS, sequentially replenishing deeper and deeper fissures. The algorithm used a multiple-pass approach rather than a recursive approach to avoid memory overflow errors. This algorithm was capable of replenishing fissures that extend relatively deep into the SMS.

### Analogue is local based

Cells within biological systems evaluate their surroundings through direct interaction with their environment. Most information is transmitted through diffusible signals (which can travel long distances but require contact with a receptor to be recognized), cell environment, or cell-cell interactions. In order to mimic that important biological reality and to preserve a clean separation between mechanism and phenomena we added a key targeted attribute to the list: CELLS must use only local mechanisms (the third attribute in Table 1). Each CELL can query the level of NUTRIENT in its local neighborhood along with the characteristics of each neighboring location. In order to locally control SMS surface irregularities and prevent fissure formation the STRESS based movement and proliferation algorithms were developed. They only required CELLS to query their immediate neighbors. Requiring that all mechanisms must be a consequence of local events would falsify some existing individual based models of tumor spheroid growth, such as [38], where an artificial gradient toward the center of the simulated tumor spheroid is created that uses global knowledge.

### Initial analogue state

Execution begins with a single CELL that grows unrestricted to 50 CELLS. At that stage, the AXIOMS are implemented. This simplification creates a slight, negligible shift in the growth curves. It should be noted that the initial growth rate of EMT6 spheroids was not clear because EMT6 cells were first grown in a monolayer, and then transferred to cultures dishes until cluster size reached about 400 cells. Only then were they placed in the spinner flasks [13].

### CELLS in context

CELLS follow axiomatic operating principles that determine state change, movement, proliferation, and resource consumption. The flow chart in Additional file 1, Fig. S4 demonstrates the full range of actions CELLS can take. CELLS exist in three states: PROLIFERATING, QUIESCENT, and NECROTIC. CELL state is determined by the amount of NUTRIENT to which CELLS are exposed. PROLIFERATING and QUIESCENT CELLS consume NUTRIENT equal to *proConsumeRate *and *quiConsumeRate*, while NECROTIC CELLS do not consume NUTRIENT.

Within a simulation cycle, when a CELL finds itself adjacent to an empty space, it will move into that space, simulating random cell movement and churning. That action has the net effect of causing spaces vacated by NECROTIC CELLS to move randomly within the SMS, eventually merging with the external space. The process is illustrated in Additional file 1, Fig. S5. When a CELL is on the outer edge, it will stochastically determine if it moves into an adjacent internal space. The probability of doing so is adjusted based on the CELL'S STRESS and is biased by the value of *moveEmptyBias*. The higher the STRESS, the greater the likelihood the CELL will move into the empty space, smoothing the local SMS edge. An outside CELL adjacent to an interior space will move into an adjacent space if PRN < p_m_. The PRN is drawn from [0,1), and p_m _is specified by an empirically derived exponential function of *moveEmptyBias *and STRESS, as detailed in Additional file 1. At constant STRESS, increasing *moveEmptyBias *decreases the likelihood of movement, while if *moveEmptyBias *is constant, increased STRESS will increase the likelihood of movement.

PROLIFERATING CELLS decrement *prolifCounter *during each cycle. When this value drops below zero, the CELL will have an opportunity to create a new CELL. If the CELL is adjacent to empty spaces, it will select one randomly, and then be given an opportunity to place a daughter CELL (a copy) at that location. A CELL given an opportunity to proliferate will do so if PRN < p_b_. The PRN is drawn from [0,1), and p_b _is specified by an empirically derived exponential function of *proBias *and STRESS, as detailed in Additional file 1. At constant STRESS, the likelihood of proliferation will decrease as *proBias *is increased, and at constant *proBias *increasing STRESS causes qualified CELLS to be less likely to proliferate.

After an attempt at creating a new CELL, *prolifCounter *is reset regardless of whether or not the attempt was successful. A daughter CELL has the same parameter values as the parent, except for *prolifCounter *and *removeCounter*, which are set to unique random values. For simplicity, we specify that CELLS are subject to contact inhibition: only CELLS adjacent to empty space can create new CELLS. Although it is not clear to what extent contact inhibition occurs *in vitro*, LaRue et al. [14] observed that only the outer two or three cell layers proliferate at the same rate as exponentially growing cells. Proliferation beyond the SMS surface was not necessary to achieve the targeted attributes (i.e., to survive falsification with the current set of targeted attributes).

### METABOLISM requires a single source of NUTRIENT

An SMS differs significantly from the individual based models of Chignola et al. [10] and Schaller et al. [6], especially in its simple representation of metabolism. The main similarities are that SMS use a diffusible NUTRIENT and CELLS DIE when insufficient NUTRIENT is available. There was no need to represent a particular type of metabolism (aerobic or anaerobic), only that CELLS consume NUTRIENT equal to *proConsume *or *quiConsume *and change state based on NUTRIENT level. We achieved the targeted attributes without being forced to add additional METABOLIC complexity. Because we achieved those attributes using a simple representation, we can achieve the same behaviors using a more complicated representation of metabolism.

### Fissure formation is related to STRESS

Early SMS versions had no means to control shape, either directly or indirectly, leading to fissure formation similar to that seen in [39]. SMS fissures were induced by diffusion-limited aggregation (DLA). DLA is a phenomenon that occurs when objects move randomly in space until they encounter and adhere to each other, forming structures with crystalline appearance [40]. SMS fissures form as the empty spaces move about because of CELL movement, and adhere when another space is encountered. Empty spaces, even though they are not actively moving objects, are subject to DLA rules because they effectively walk randomly through the SMS. Fissures form as spaces connect to each other. The inner extreme of a fissure is the closest outside spaces to the NECROTIC core, where spaces are generated. In order to prevent fissure formation, we developed the STRESS-based proliferation algorithm. It helps prevent fissure development. CELLS at the inner extreme of any fissure will have low STRESS values, leading to preferential proliferation at that location.

**Figure 13:**
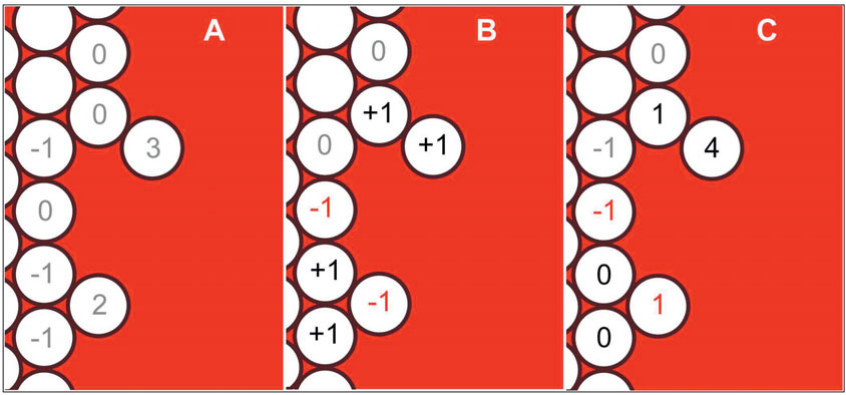
**Illustration of a CELL determining its level of STRESS**. (A) *InitialStress *is calculated based on the number of empty spaces. (B) The change in STRESS is calculated based on number of outside neighbors and their *initialStress *values, with some CELLS increasing in STRESS (black values), some decreasing (red values) and others staying the same (gray values). (C) STRESS is calculated by summing the value of *initialStress *and the change in the value of STRESS.

**Figure 14:**
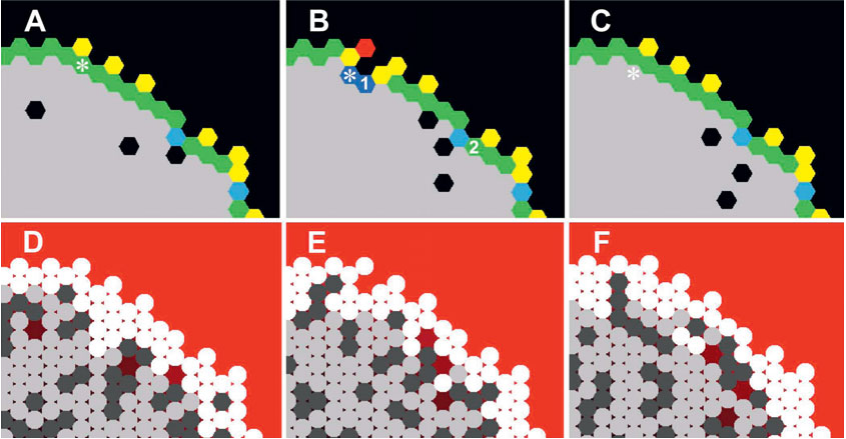
**Illustration of CELLS responding to STRESS at low NUTRIENT**. (A-C) Illustrations of STRESS levels at sequential time steps. Only CELLS at the surface are color-coded. STRESS levels: dark blue = -2, light blue = -1, green = 0, yellow = 1, orange = 2, and red = 3. (A) During one simulation cycle, the empty space below and to the left of the starred (*) CELL is adjacent to that CELL; the CELL then moves inward to fill that empty space. (B) During the next simulation cycle, the starred CELL has a low STRESS and so becomes likely to create a new CELL. The stress algorithm allows CELLS that have equivalent immediate neighborhoods, such as the CELLS labeled 1 and 2, to have different STRESS values. Because the neighbors of CELL 1 have higher *initialStress *values than the neighbors of CELL 2, CELL 1 will have a higher STRESS and be more likely to create a new CELL during the simulation. (C) During the third simulation cycle the starred CELL creates a new CELL, places it in the adjacent space, resulting in a return to initial conditions. (D-F): CELL state view at equivalent TIME steps.

In order to avoid extreme, abiotic SMS surface irregularities, each CELL creates new CELLS and moves based on its STRESS value. STRESS maps somewhat to the adhesion mechanisms used in [2,6]. It also maps to a combination of surface tension and adhesion. However, it was only necessary to have it operate at the SMS surface. CELLS having smaller STRESS values will be more likely to create new CELLS and less likely to move inward into empty spaces. To calculate STRESS, a CELL uses a two-pass algorithm in each cycle. First, it determines its *initialStress*: it subtracts two from the number of empty spaces in the neighborhood. During the second pass, a CELL counts the number of CELLS in its neighborhood that are on the SMS edge (*outsideNeighbors*). As illustrated in Fig. 13, CELLS then calculate their final stress depending on *outsideNeighbors*. If *outsideNeighbors ≠ *2, *finalStress *= *initialStress *+ 1. If *outsideNeighbors *= 2, the CELL queries these two neighbors and sums their *initialStress*, and if that sum < 0, *finalStress *= *initialStress *- 1. If the sum is > 1, *finalStress *= *initialStress *+ 1, and if the sum is 0 or 1, *finalStress *= *initialStress*. This algorithm has the effect of transmitting the STRESS felt by one CELL to its neighbors, enabling CELLS to have different final STRESS values even if their neighborhoods are identical. Figure 14 shows sequential screen shots of the stress felt by CELLS in a growing SMS and demonstrates the preferential nature of proliferation. Figure 14a shows the initial arrangement. In Fig. 14b, the starred CELL has moved to fill an empty space, changing the local CELL arrangement and each CELL'S resulting STRESS value. Consequently, the starred CELL has a very low STRESS value and a corresponding higher chance of proliferating. When its *prolifCounter *reaches zero, the starred CELL creates a new CELL, as illustrated in Fig. 14c, returning that portion of the SMS to its original arrangement.

### Shedding of cells from the SMS surface

An occasional CELL will become isolated near the SMS surface because of normal AXIOM operation. In order to prevent their local accumulation, we implemented an algorithm that simulates shedding and the consequences of shear force caused by stirring. Any CELL that has no CELL neighbors will move randomly, selecting one of its six immediate neighbors using a uniform distribution, stopping when it encounters another CELL. Most CELLS reattach to the SMS or form small clusters. An occasional CELL will move far enough to exit the grid; it is then removed from the simulation. The number of CELLS shed currently is significantly smaller than that reported in [20]. Shedding was not deemed a sufficiently important attribute to target at this stage. Should it be targeted, it will be straightforward to add a shedding AXIOM and then adjust parameter values to reestablish matching behaviors. As an example, a similar modification was carried out between an earlier version of the analogue [41] and the current one. Cells in [41] consumed the same quantity of NUTRIENT regardless if they were in the QUIESCENT or PROLIFERATING state, but in the current analogue the amount consumed is different.

## Authors' contributions

JAE and CAH conceived and designed the analogue. JAE implemented the simulation with assistance from GEPR, and performed the experiments. JAE, CAH, and GEPR analyzed the data and refined the analogue. JAE and CAH wrote the paper with input from GEPR.

## Supplementary Material

Additional file 1 **Supplementary information.** This PDF file contains supplementary figures explaining additional results and methods, as well as a detailed explanation of the CELL movement and proliferation algorithms. Click here for file
