# Supplementary material for: Essential operating principles for tumor spheroid growth
Source: BMC Syst Biol. 2008 Dec 23;2:110. doi: 10.1186/1752-0509-2-110 (PMC2667182; doi:10.1186/1752-0509-2-110)
Supplement: Additional file 1 — Supplementary information. This PDF file contains supplementary figures explaining additional results and methods, as well as a detailed explanation of the CELL movement and proliferation algorithms. [file 1752-0509-2-110-S1.pdf]

Supplementary information:

Essential operating principles for tumor spheroid growth

Jesse A. Engelberg, Glen E. P. Ropella, C. Anthony Hunt

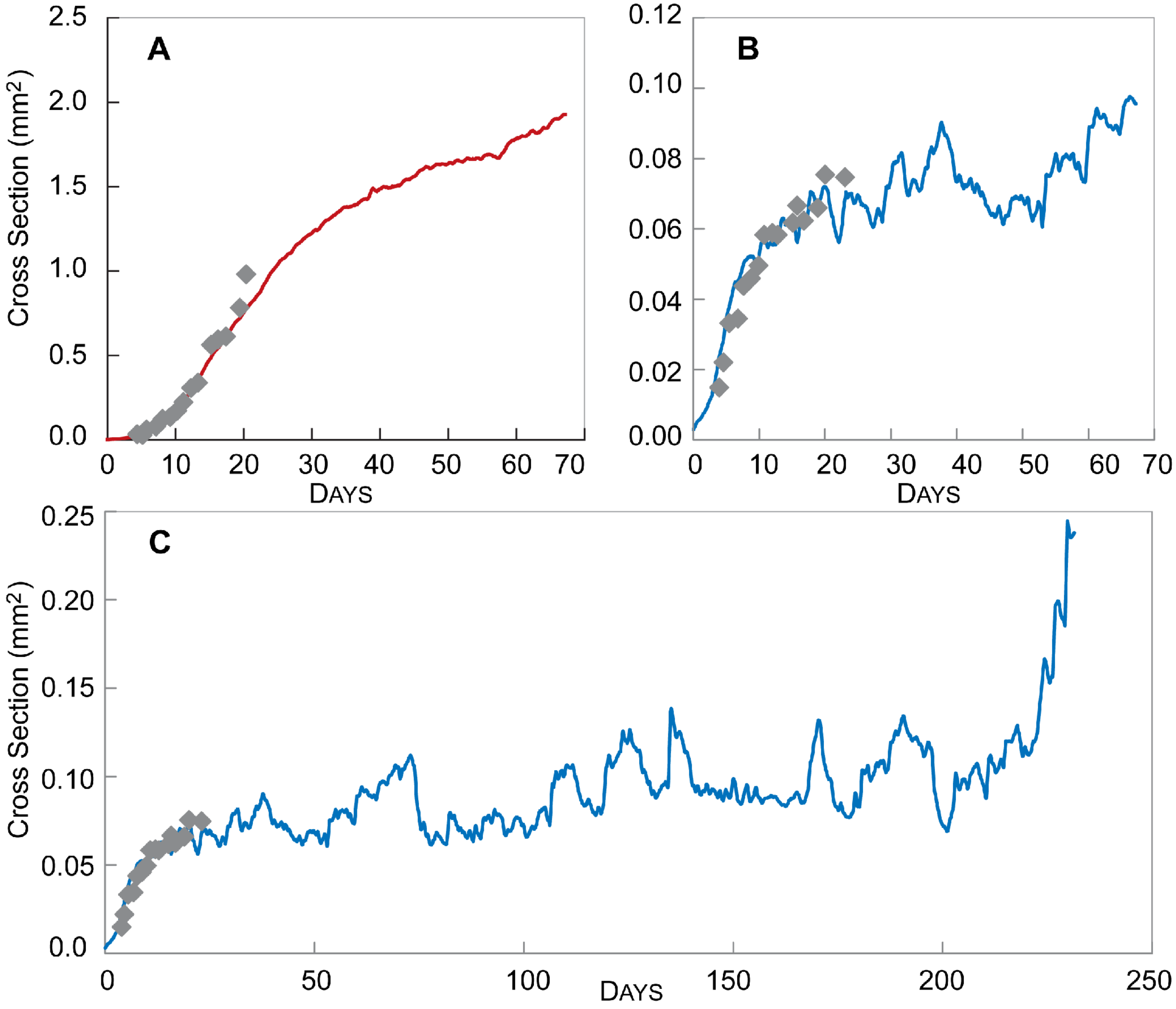

Figure S1. Results from three extended SMS experiments. Gray circles: *in vitro* data as in Fig. 3. Parameter values were those listed in Table 3. (A) SMS growth at high NUTRIENT for 67 DAYS. SMS growth at low NUTRIENT for 67 (B) and 230 (C) DAYS. Eventual maximum size before destabilization for both (A)

and (B) is similar to that predicted by fitting the growth of multiple runs to the Gompertz equation, as observed in Table 4. In (C) the SMS remains stable in size for an extended period of time, but eventually destabilizes.

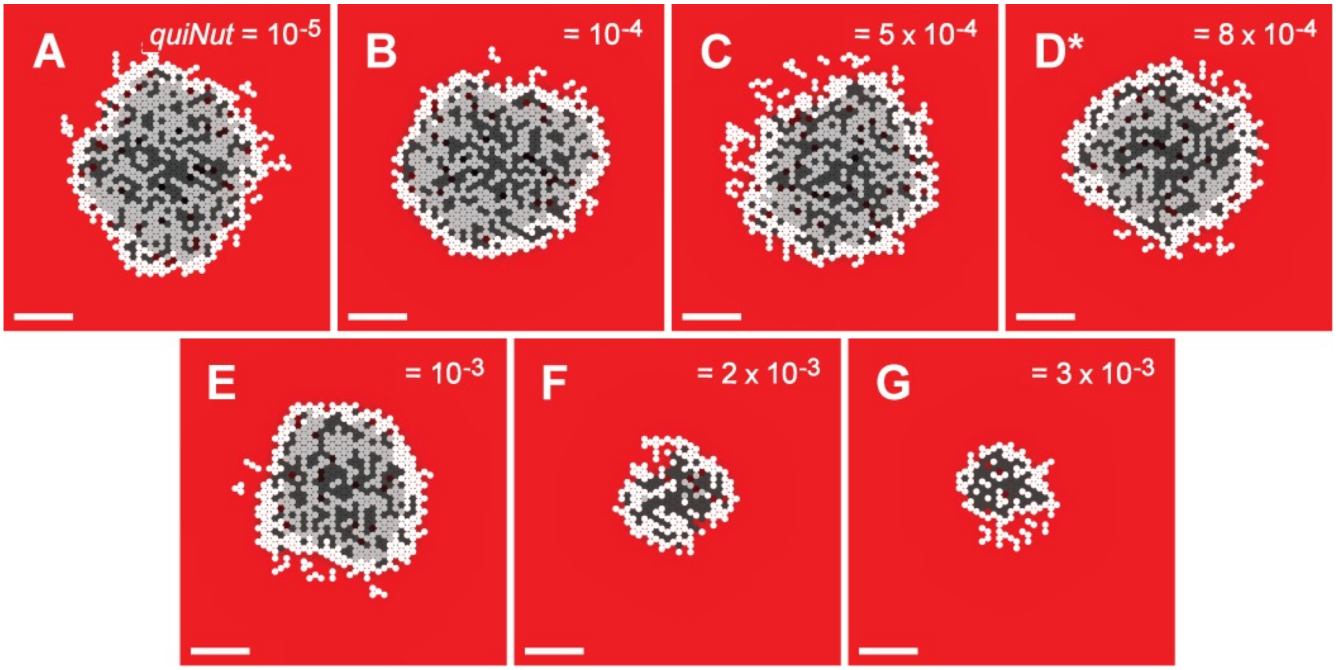

Figure S2. SMS cross sections at varied *quiNut* values and low NUTRIENT. All images were recorded at 21 DAYS. Scale bar: 100 µm. Except for *quiNut*, parameter values were those listed in Table 3. (A)-(G): *quiNut* values are shown. Stabilization size and VIABLE rim widths decreased as *quiNut* increased, but all experiments achieved stability. *: *quiNut* value in Table 2.

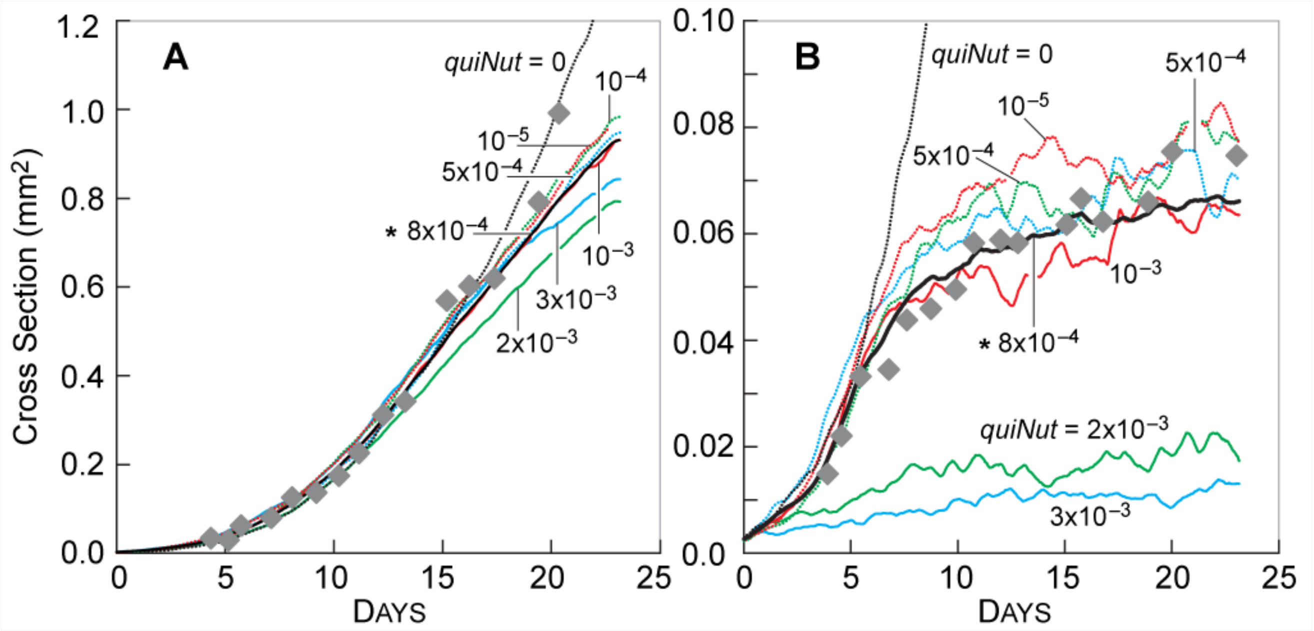

Figure S3. Influence of quiNut on SMS growth. Gray circles: in vitro data as in Fig. 3. Except for quiNut, parameter values were those listed in Table 2. Colored lines are results of single experiments for the indicated values of quiNut from 0 to 3.0 x 10^{-3} (same values as in Fig. S2). (A) high NUTRIENT; (B) low NUTRIENT. *: quiNut value in Table 2.

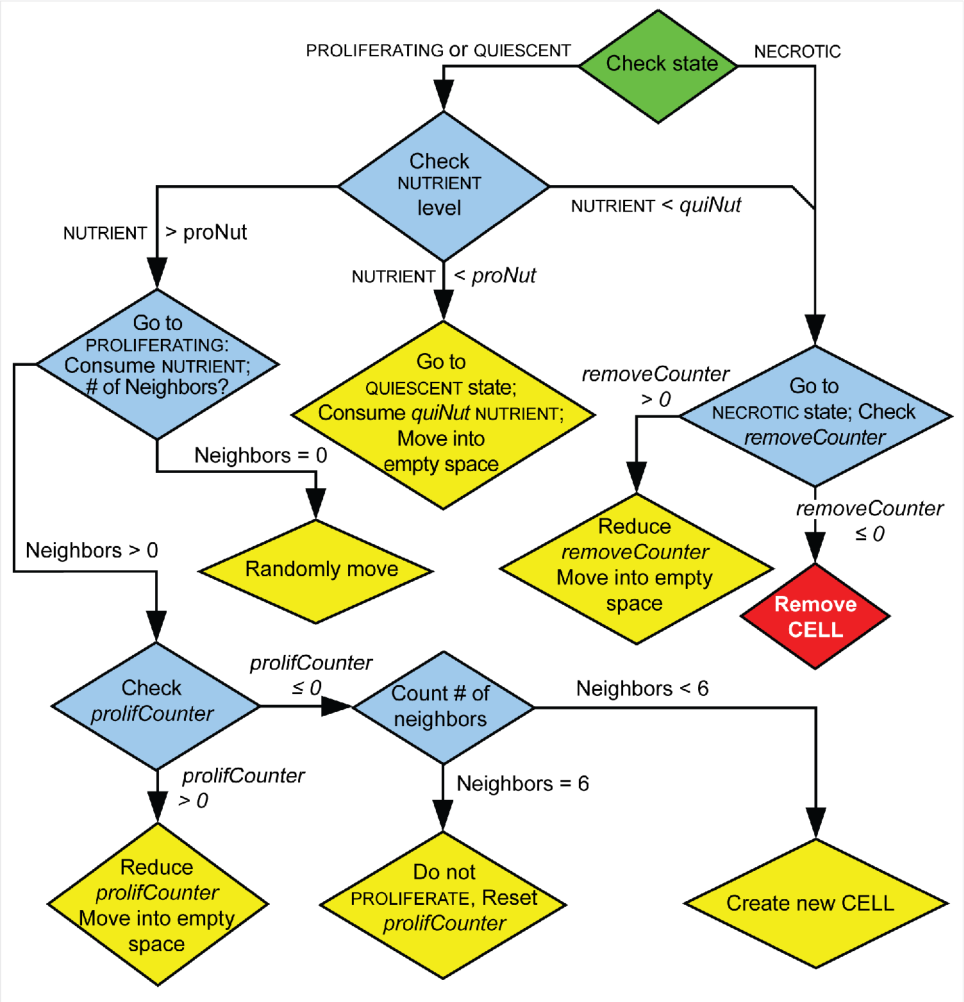

Figure S4. Flow chart of action options for an individual CELL. During each simulation cycle, a CELL begins by checking its state (green diamond). It then checks the conditions of its local environment, as well as values of internal variables to determine intermediate actions (blue diamonds), final actions (yellow diamonds) or CELL death (red diamond). CELLS that move into an empty space will do so deterministically if they are inside the border of the SMS, but stochastically if they are on the outside of the SMS. CELLS that have no neighbors will randomly move outside of the SMS.

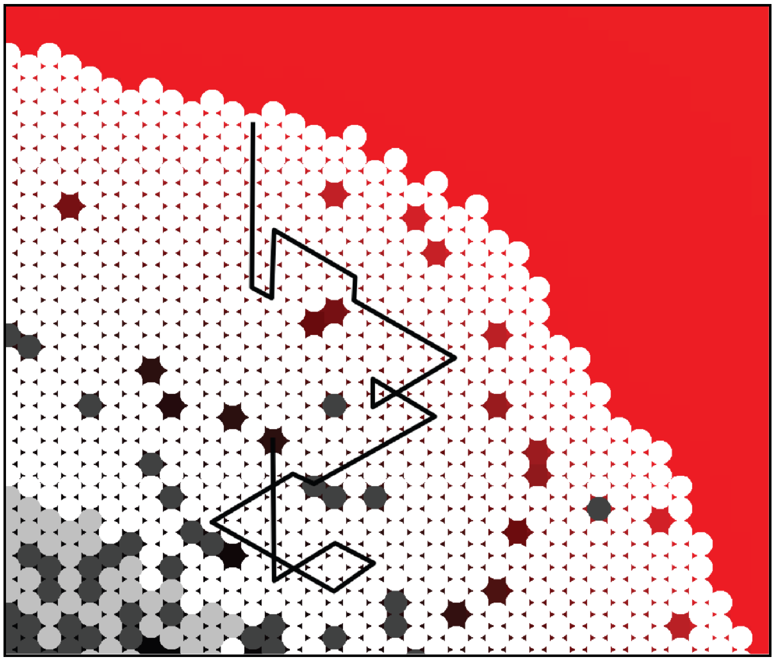

Figure S5. Relocation of empty spaces. Random CELL movement into an adjacent empty space produces the net movement of spaces over time. During each simulation cycle, any CELL adjacent to an internal empty space move into that space. Consequently, empty spaces move randomly (black line) within the SMS until they exit at the outside edge. Spaces may be moved multiple times during a single simulation cycle.

Stochastic cell movement and proliferation

When a CELL on the outside of the SMS is adjacent to an empty space it will move into that space if a PRN < p_m, where PRN is a pseudo random number in the interval [0,1). The value of p_m is determined by the function $p_m = \left(\frac{S+3}{6}\right)^{7m+1}$, where S is the STRESS experienced by the CELL and m is the value of *moveEmptyBias*. This function has the effect of skewing the distribution of p_m based on *moveEmptyBias*. The higher the value of *moveEmptyBias* the more extreme the skewing will be, so that CELLS with a low STRESS and a high *moveEmptyBias* will almost never move into adjacent empty spaces, while CELLS with a high STRESS will do so regardless of the value of *moveEmptyBias*.

CELLULAR proliferation is biased in a similar fashion, except that in this case CELLS with a high STRESS will be less likely to create new CELLS. CELLS will proliferate if PRN < p_b, with PRN being between [0,1). The equation used to determine the value of p_b is $p_b = (\frac{6-S}{8})^{7b+1}$, with S being the STRESS of the CELL and b being the value of *proBias*. The skewing effect is similar, but the influence of STRESS is reversed, so CELLS with a high STRESS and high *proBias* will almost never create new CELLS, while CELLS with low STRESS will be very likely to create new CELLS regardless of the value of *proBias*.
